# The Shiga Toxin Receptor Globotriaosylceramide as Therapeutic Target in Shiga Toxin *E. coli* Mediated HUS

**DOI:** 10.3390/microorganisms9102157

**Published:** 2021-10-16

**Authors:** Wouter J. C. Feitz, Romy Bouwmeester, Thea J. A. M. van der Velden, Susan Goorden, Christoph Licht, Lambert P. J. W. van den Heuvel, Nicole C. A. J. van de Kar

**Affiliations:** 1 Department of Pediatrics, Amalia Children’s Hospital, Radboudumc, 6525 GA Nijmegen, The Netherlands; wouter.feitz@radboudumc.nl (W.J.C.F.); romy.bouwmeester@radboudumc.nl (R.B.); thea.vandervelden@radboudumc.nl (T.J.A.M.v.d.V.); bert.vandenheuvel@radboudumc.nl (L.P.J.W.v.d.H.); 2 Department of Pediatric Nephrology, Amalia Children’s Hospital, Radboud Institute for Molecular Life Sciences, Radboudumc, 6525 GA Nijmegen, The Netherlands; 3 Laboratory Genetic Metabolic Diseases, Department of Clinical Chemistry, Amsterdam UMC, University of Amsterdam, 1105 AZ Amsterdam, The Netherlands; s.m.goorden@amsterdamumc.nl; 4 Department of Pediatric Nephrology, The Hospital for Sick Children, Toronto, ON M5G 1X8, Canada; Christoph.licht@sickkids.ca; 5 Department of Pediatric Nephrology, Academic Hospitals Leuven, 3000 Leuven, Belgium; 6 Department of Development and Regeneration, Academic Hospitals Leuven, 3000 Leuven, Belgium

**Keywords:** STEC-HUS, shiga toxin, endothelial cells, globotriaosylceramide, glucosylceramide synthase, eliglustat

## Abstract

In 90% of the cases, childhood hemolytic uremic syndrome (HUS) is caused by an infection with the Shiga toxin (Stx) producing *E. coli* bacteria (STEC-HUS). Stx preferentially binds to its receptor, the glycosphingolipid, globotriaosylceramide (Gb3), present on the surface of human kidney cells and various organs. In this study, the glycosphingolipid pathway in endothelial cells was explored as therapeutic target for STEC-HUS. Primary human glomerular microvascular endothelial cells (HGMVECs) and human blood outgrowth endothelial cells (BOECs) in quiescent and activated state were pre-incubated with Eliglustat (Cerdelga^®^; glucosylceramide synthase inhibitor) or Agalsidase alpha (Replagal^®^; human cell derived alpha-galactosidase) in combination with various concentrations of Stx2a. Preincubation of endothelial cells with Agalsidase resulted in an increase of α-galactosidase activity in the cell, but had no effect on the binding of Stx to the cell surface when compared to control cells. However, the incubation of both types of endothelial cells incubated with or without the pro-inflammatory cytokine TNFα in combination with Eliglustat resulted in significant decrease of Stx binding to the cell surface, a decrease in protein synthesis by Stx2a, and diminished cellular Gb3 levels as compared to control cells. In conclusion, inhibition of the synthesis of Gb3 may be a potential future therapeutic target to protect against (further) endothelial damage caused by Stx.

## 1. Introduction

The most frequent form of HUS in childhood is caused by a gastro-intestinal infection with the Shiga toxin (Stx) producing *E. coli* bacteria (STEC) [1]. For STEC-HUS, characterized by thrombocytopenia, nonimmune hemolytic anemia, and acute kidney failure [2], no specific therapy is yet available [3,4]. Patients are treated with supportive therapy alone. The disease can lead to end stage renal disease (ESRD) and has a mortality rate of 2–5% in the acute phase [5]. Long-term renal sequelae like hypertension, proteinuria, and chronic kidney failure are reported in an estimated 25–30% of cases [5].

After intestinal colonization by STEC, Stx is released into the intestinal lumen and translocated to the blood circulation. Various studies have shown that the toxin might bind to blood cells (i.e., leukocytes, red blood cells, and/or platelets) and/or to microvesicles that originate from those blood cells [6,7,8,9]. Bound Stx travels to its principal receptor globotriaosylceramide (Gb3), located on the surface of various cells including erythrocytes, brain endothelial cells, and human kidney cells like glomerular endothelial cells and podocytes [10,11,12,13,14,15,16]. Uptake by endocytosis takes place, leading to protein synthesis inhibition and cell death [17].

**Figure 1:**
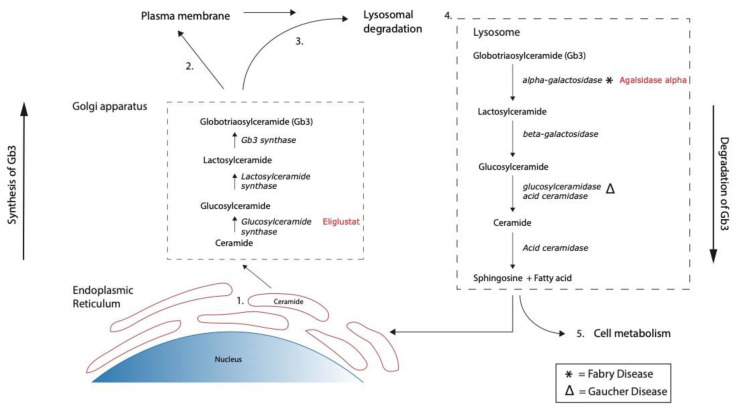
Schematic representation of the synthesis from ceramide to globotriaosylceramide (Gb3) and the breakdown of Gb3 to ceramide with the different specific enzymes involved. Ceramide travels from the endoplasmic reticulum to the Golgi apparatus, where a plurality of specific enzymes regulates further metabolism (**1**). After synthesis, Gb3 travels to the plasma membrane, where it resides (**2**) or travels to lysosomes for degradation by specific enzymes (**3**,**4**). Breakdown products are re-used for glycosphingolipid synthesis (**5**) or function in cell metabolism processes. * and ∆ refer to the enzymes affected in Fabry disease (deficiency of alpha-galactosidase) and Gaucher disease (deficiency of glucosylceramidase), respectively. Eliglustat inhibits glucosylceramide synthase, while Agalsidase alpha substitutes alpha-galactosidase.

Gb3 belongs to the group of (glyco)sphingolipids. They are structurally and functionally diverse molecules important for cell homeostasis [18]. Next to the modulation of cell-cell interactions, they are able to regulate differentiation, proliferation, and programmed cell death. The backbone of all complex glycosphingolipids is the lipid moiety ceramide [19]. Generation of ceramide takes place de novo in the endoplasmic reticulum (ER) and a plurality of glycosyltransferases and glycosidases are involved in the synthesis towards Gb3, and vice versa for the breakdown of Gb3 into ceramide [19] (Figure 1).

Gb3 has not only a central role in the pathophysiology of STEC-HUS, but it also plays a pivotal role in Fabry disease. Fabry disease is a X-linked lysosomal storage disorder with pathogenic accumulation of Gb3 caused by deficiency of the enzyme alpha-galactosidase [20]. Treatment of Fabry disease focuses on replacement of alpha-galactosidase activity by substitution of the enzyme with as main principle increased breakdown of Gb3, leading to reduced storage inside the cell. Substitution of human alpha-galactosidase is clinically available as enzyme replacement therapy (ERT) and has shown clinical benefit in a subset of patients [21] (Figure 1). In addition, Gaucher disease is a rare autosomal recessive lipid storage disease caused by deficiency of glucosylceramidase (also referred to as glucocerebrosidase), resulting in accumulation of glucosylceramide, one of the precursors of Gb3 [22]. Patients with Gaucher disease are (besides ERT) treated with so-called substrate reduction therapy (SRT) using inhibitors of glucosylceramide synthase [23,24] (Figure 1).

Enzyme replacement therapies as well as substrate reduction therapies might be of interest in the treatment of STEC-HUS as Gb3 plays an important role in the pathophysiology. Recent in vitro studies have shown the ability of the substrate reduction inhibitor Eliglustat (Cerdelga^®^) to decrease the sensitivity for Stx in human colonic epithelial cells and human renal tubular epithelial cells [25,26]. Eliglustat inhibits glucosylceramide synthase activity and, thus, de novo formation of Gb3 (Figure 1) [23]. The gastro-intestinal tract and kidneys are the main affected organs in STEC-HUS with acute renal failure as one of the symptoms due to the thrombotic microangiopathic processes seen in the glomeruli of the kidney. The purpose of this research was the investigation of the effect of Eliglustat and Agalsidase alpha (Replagal^®^, a human derived alpha galactosidase compound that stimulates breakdown of Gb3 in the cell) on human (glomerular) endothelial cells and the consequences for the interactions with Stx.

Primary human glomerular microvascular endothelial cells (HGMVECs) and human blood outgrowth endothelial cells (BOECs) were used in our studies. The effect of Eliglustat and Agalsidase alpha on cellular Gb3 levels, Stx binding to its receptor Gb3 on the cell surface, and the effect of Stx on protein synthesis was examined with liquid chromatography-mass spectrometry (LC-MS), flow cytometry, and a protein synthesis assay.

## 2. Materials and Methods

### 2.1. Cell Culture 

Primary human glomerular microvascular endothelial cells (HGMVECs) from four healthy adult donors were collected. The isolation and purification of HGMVECs was carried out as previously described [15]. Cells with 80–100% confluency, passage 5–11, were used for experiments and grown in 1% gelatin/distilled water (Sigma Aldrich, St. Louis, MI, USA) coated cell culture plates (Corning Incorporated, Kennebunk, ME, USA). Human blood outgrowth endothelial cells (BOECs) from three healthy adult donors were isolated and characterized according to established procedures [27,28]. Cells with 80–100% confluency, passage 4–6, were used for experiments and grown in cell culture plates coated with collagen type 1 (Corning Discovery Labware, Bedford, MA, USA).

### 2.2. Reagents 

Shiga toxin subtype 2a (Stx2a) was ordered from Phoenix Lab (Tufts Medical Center, Boston, MA, USA). The amount of LPS remaining in these preparations was determined to be less than 0.10 U/mL. Alexa 488 labeled Shiga toxin subunit B (Stx-B) was provided by C. Lingwood (The Hospital for Sick Children, Toronto, ON, Canada). Agalsidase alpha (Replagal^®^) was a gift from C. Hollak (Amsterdam Medical Centre, Amsterdam, The Netherlands). Eliglustat hemitartrate was ordered from Cayman Chemicals (Ann Arbor, MI, USA).

### 2.3. Immunofluorescence Staining and Imaging of Bound Shiga Toxin Subunit B and CD-31

HGMVECs and BOECs were seeded in a black 96-well plate with a clear bottom (Falcon Corning Incorporated, Corning, NY, USA). Cells were incubated with either control medium or medium with 10 ng/mL of TNFα (Roche Diagnostics GmbH, Mannheim, Germany) for 24 h and fixed with 2% paraformaldehyde (PFA)/PBS (Sigma Aldrich) at room temperature for 15 min. Mouse anti-CD31 (BD Biosciences, San Jose, CA, USA) primary and corresponding species-specific Alexa Fluor 568 were used as antibodies. Alexa 488-labeled Stx-B at a concentration of 5 μg/mL and the fluorescent DNA stain DAPI (Invitrogen, Carlsbad, CA, USA) were added, and images were taken with the use of a Zeiss LSM900 confocal microscope (Zeiss, Oberkochen, Baden-Württemberg, Germany).

### 2.4. Analysis of Shiga Toxin Subunit B Binding by Flow Cytometry 

HGMVECs and BOECs were pre-incubated with or without 10 ng/mL of TNFα, 8.0 nM of Eliglustat, or 10 μg/mL of Agalsidase alpha for 24 h. After pre-incubation, cells were fixed with 0.5% PFA/PBS for 5 min at room temperature. Next, cells were incubated with Alexa 488-labeled Stx-B at a concentration of 5 μg/mL and stored for 30 min at 4 °C, protected from light. After 30 min., cells were trypsinized with 0.05% Trypsin-EDTA (Gibco Thermo Fisher Scientific, Waltham, MA, USA), suspended in 10% FBS/PBS (Gibco Thermo Fisher Scientific), spun down by centrifugation, and resuspended in 0.1% BSA/PBS (Sigma Aldrich). Read out was done with the use of a CytoFlex Flow Cytometer (Beckman Coulter, Brea, CA, USA). 

### 2.5. Protein Synthesis by Radiolabeled ^3^H-Leucine Incorporation Assay 

HGMVECs and BOECs were pre-incubated with or without TNFα, Eliglustat, or Agalsidase alpha as described above. After 24 h, Stx2a in a concentration of 1 nM or 10 nM together with ^3^H-leucine (PerkinElmer, Boston, MA, USA) for 24 h was added. In another condition, cells were pre-incubated with or without TNFα for 24 h. Next, cells were incubated with Eliglustat together with Stx2a and ^3^H-leucine at the same time for 24 h. The ^3^H-leucine incorporation assay was performed as established in the past [28].

### 2.6. Cellular Gb3 and Ceramide Levels by Liquid Chromatography-Mass Spectrometry

HGMVECs and BOECs were pre-incubated with or without 10 ng/mL of TNFα, 8.0 nM of Eliglustat, or 10 μg/mL of Agalsidase alpha for 24 h. After incubation, cell pellets were collected and stored at −80 °C until further use. The analysis of cellular Gb3 and ceramide levels was performed with liquid chromatography-mass spectrometry (LC-MS). Sphingosine and d7-sphingosine were obtained from Avanti Polar Lipids Inc. (Alabaster, AL, USA) and all organic solvents were obtained from Biosolve (Valkenswaard, The Netherlands). Formic acid, butanol, and hydrochloric acid were obtained from Merck (Darmstadt, Germany), and sodium hydroxide and ammonium formate were from Sigma Aldrich. Cell pellets were dissolved in 200 µL water and sonicated at 8.5-Watt output for 7 s. The protein concentration was determined using a BCA-based assay. The metabolites were extracted from cell homogenates by a modification of the method of Bligh and Dyer [29]. Briefly, 600 µL methanol, 600 µL chloroform, and 450 µL water was added to 150 µL homogenate. Next, the lower phase (500 µL) was transferred to a microwave tube and the upper phase was washed with chloroform. The lower phase was added to the first and the combined phases were dried under a stream of nitrogen at 40 °C. The residue was dissolved in 500 µL 0.1 M sodiumhydroxide in methanol and samples were deacylated [30]. After this, samples were neutralized by addition of 50 µL 0.1 M hydrochloric acid, internal standards (25 µL of 1 µM d7-sphingosine in methanol) were added, and samples were dried and dissolved in butanol and water (1:1, *v/v*). After drying the butanol phase, samples were dissolved in 120 µL methanol and analyzed by LC-MS/MS. Metabolites were calculated using calibration lines within the appropriate concentration range, according to the internal standard ratio method. Metabolites were separated by RP-UPLC using an Acquity I-Class UPLC with BEH C18 column, 2.1 × 50 mm with 1.7 μm particle size (Waters Inc., Milford, MA, USA), and detected by electrospray ionization in positive mode (ESI+) and MS/MS-instrument (Xevo TQ MS, Waters Inc., Milford, MA, USA) in multiple reaction monitoring (MRM) mode. The different glycosphingolipid species were measured after deacylation and did not contain a fatty acid anymore. A sum of all isoforms with a d18:1 and d18:2 base regardless of the fatty acid was measured and noted in this study as d18:1/X and d18:2/X. For details on the LC-MS/MS methods and settings used, see [31].

### 2.7. Statistical Analysis

Data were analyzed by unpaired Student’s *t*-test or ANOVA. A *p*-value of <0.05 was set as statistically significant. All statistical analyses were performed using GraphPad Prism version 5.03 (GraphPad Software, La Jolla, CA, USA) or SPSS (IBM, Amsterdam, The Netherlands). Data are expressed as mean ± SEM.

## 3. Results

### 3.1. The Effect of Eliglustat and Agalsidase Alpha on Shiga Toxin Binding to the Endothelial Cell Surface

**Figure 2:**
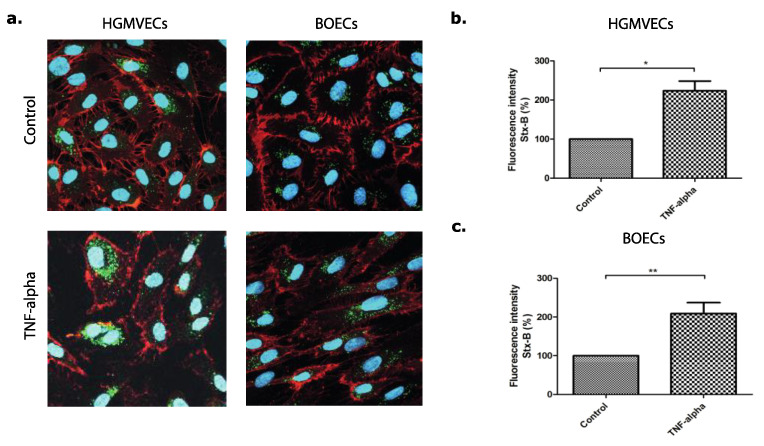
The expression and binding of Alexa 488 labelled Shiga toxin subunit B (Stx-B) on the cell surface of primary HGMVECs and BOECs. (**a**) Immunofluorescent images of Stx-B on HGMVECs and BOECs pre-incubated without (control) or with 10 ng/mL of TNFα for 24 h. Stx-B in green and CD-31 in red. The nucleus is stained in blue; 400× Magnification by confocal microscopy. Flow cytometry of the binding of Stx-B on the cell surface of (**b**) primary HGMVECs or (**c**) BOECs pre-incubated without (control) or with 10 ng/mL of TNFα for 24 h. Pre-incubation of TNFα resulted in an increase of Stx-B binding on the endothelial cell surface of both HGMVECs and BOECs. Statistically significant differences are indicated with single (*p* < 0.05) or double (*p* < 0.01) characters, respectively.

Binding of Stx to the endothelial cell surface was determined by flow cytometry in combination with fluorescent labelled Shiga toxin subunit B (Stx-B) in order to indirectly measure Gb3 cell surface expression levels (20). TNFα was used as positive control, as TNFα is a pro-inflammatory cytokine known to upregulate Gb3 expression on the cell surface by increasing the activity of enzymes involved in the Gb3 precursor synthesis (Figure 2) [32,33]. 

**Figure 3:**
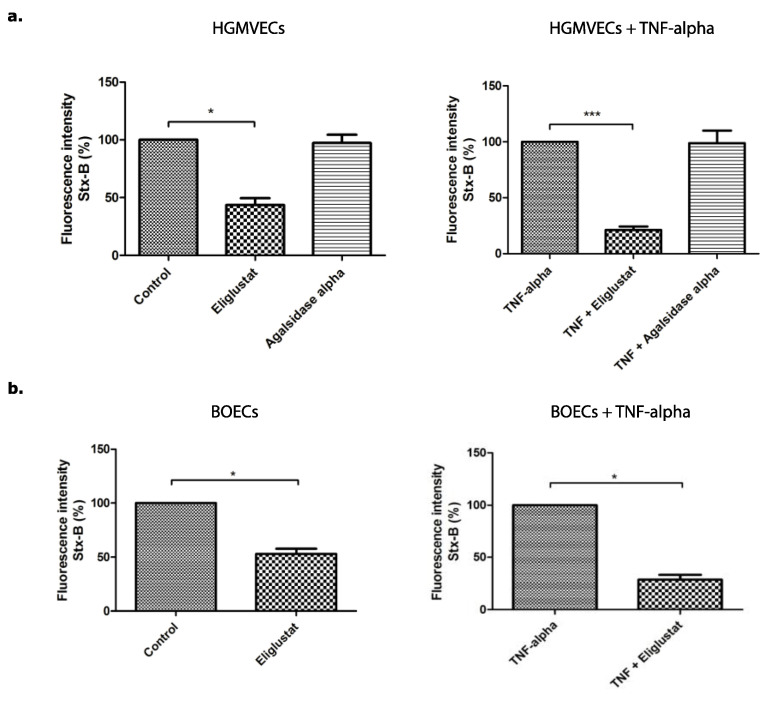
The binding of Alexa 488 labelled Stx-B to the cell surface of primary HGMVECs or BOECs. Binding of Stx-B to the cell surface of (**a**) HGMVECs or (**b**) BOECs incubated with or without TNFα in combination with or without 8.0 nM Eliglustat or 10 μg/mL of Agalsidase alpha for 24 h. Read out performed with the use of flow cytometry. Statistically significant differences are indicated with single (*p* < 0.05) or triple (*p* < 0.001) characters, respectively.

Primary HGMVECs from four healthy donors were incubated with or without 10 ng/mL TNFα, 8.0 nM of Eliglustat, or 10 μg/mL of Agalsidase alpha for 24 h. The concentrations and incubation times used for these experiments were determined by flow cytometry titration curves (data not shown) or established before [34]. Agalsidase alpha had no significant effect on Stx-B binding to the cellular surface, while the binding decreased by 56% in cells incubated with Eliglustat in comparison to the control group (Figure 3a). An even stronger effect was obtained when primary HGMVECs were incubated with TNFα in combination with Eliglustat. TNFα in combination with Eliglustat for 24 h resulted in a decrease of Stx-B binding by 79% as compared to the control group treated with TNFα solely (Figure 3a).

To study if the effect of Eliglustat was endothelial cell type specific, another endothelial cell type was used for these experiments. Human derived BOECs from three different donors were incubated with 8.0 nM of Eliglustat for 24 h. Incubation with Eliglustat alone resulted in a decrease of Stx-B binding on the cell surface by 47% (Figure 3b). When BOECs were incubated with 10 ng/mL of TNFα in combination with 8.0 nM of Eliglustat for 24 h, Stx-B binding decreased by approximately 72% compared to BOECs pre-incubated with 10 ng/mL of TNFα for 24 h alone (Figure 3b).

### 3.2. The Effect of Eliglustat and Agalsidase Alpha on Protein Synthesis Inhibition by Stx2a in Endothelial Cells

Uptake of Stx into the cell by endocytosis resulted in the inhibition of protein synthesis and cell death [17]. As we detected a markedly reduced Stx-B binding to the endothelial cell surface after incubation with Eliglustat, we investigated the effect of (especially) Eliglustat on endothelial protein synthesis inhibition by Stx2a.

Primary HGMVECs were pre-incubated with or without TNFα, Eliglustat, or Agalsidase alpha for 24 h. Next, cells were incubated with 1.0 or 10 nM of Stx2a for 24 h, and the effect on protein synthesis was examined with a ^3^H-leucine incorporation assay [28].

**Figure 4:**
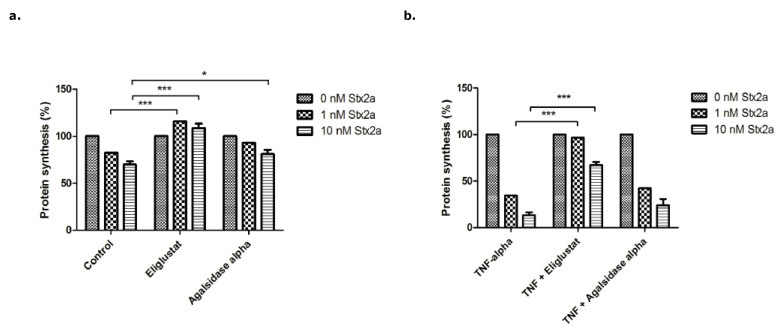
Protein synthesis of primary HGMVECs incubated with Stx2a for 24 h. (**a**) Protein synthesis of HGMVECs pre-incubated with 8.0 nM Eliglustat or 10 μg/mL Agalsidase alpha for 24 h. After 24 h, cells were incubated with Stx2a for 24 h. (**b**) Protein synthesis of HGMVECs pre-incubated with 10 ng/mL TNFα or TNFα in combination with Eliglustat or Agalsidase alpha. Statistically significant differences are indicated with single (*p* < 0.05) or triple (*p* < 0.001) characters, respectively.

Stx2a inhibited protein synthesis in HGMVECs in a concentration dependent manner with inhibition by 18% for 1.0 nM of Stx2a and by 30% for 10 nM of Stx2a (Figure 4a—Control). Pre-incubation with 10 ng/mL of TNFα for 24 h increased the inhibition of protein synthesis by Stx2a (Figure 4b—TNFα), while pre-incubation with Eliglustat protected cells from protein synthesis inhibition caused by Stx2a (Figure 4a). A marginal effect was observed with Agalsidase alpha, in contrast to Eliglustat, which displayed a clear protective effect for protein synthesis inhibition by Stx2a.

More importantly, when 10 ng/mL of TNFα at the same time as Eliglustat or Agalsidase alpha was added, Eliglustat still significantly counteracted the decreased protein synthesis caused by Stx2a (Figure 4b). Stx2a application of 1.0 nM for 24 h hardly caused any inhibition (estimated 3%) in the TNFα + Eliglustat group vs. 65% when TNFα alone was used (Figure 4b). For 10 nM Stx2a, this was 33% vs. 87%, respectively. Agalsidase alpha had no significant effect on protein synthesis caused by Stx2a when used in combination with TNFα (Figure 4b).

**Figure 5:**
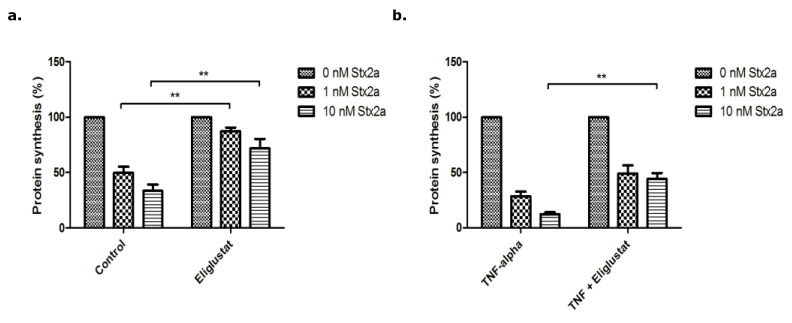
Protein synthesis of BOECs incubated with Stx2a for 24 h. (**a**) Protein synthesis of BOECs pre-incubated with 8.0 nM Eliglustat for 24 h. After 24 h, cells were incubated with Stx2a for 24 h. (**b**) Protein synthesis of BOECs pre-incubated with 10 ng/mL of TNFα or TNFα in combination with Eliglustat at the same time for 24 h. After 24 h, cells were incubated with Stx2a for 24 h. Statistical significant differences are indicated with double characters (*p*-value < 0.01).

Next, the effect of Eliglustat on protein synthesis in BOECs was investigated (Figure 5). Stx2a alone inhibited protein synthesis in a concentration dependent manner with 50% for 1.0 nM and 65% for 10 nM of Stx2a (Figure 5a—Control). Pre-incubation with 8.0 nM of Eliglustat for 24 h resulted in less protein synthesis inhibition (13%) when 1.0 nM Stx2a was used, and 28% with 10 nM of Stx2a (Figure 5a—Eliglustat). Pre-incubation of BOECs with 10 ng/mL of TNFα together with 8.0 nM of Eliglustat caused a decrease of protein synthesis by 51% for 1.0 nM Stx2a (vs. 71% for TNFα alone). A concentration of 10 nM Stx2a resulted in 56% protein synthesis inhibition (vs. 88% for TNFα alone) (Figure 5b).

In summary, Eliglustat decreased the inhibition of protein synthesis caused by Stx2a in both primary HGMVECs and BOECs.

Finally, we examined the effect of Eliglustat incubated at the same time (instead of sequentially) with Stx2a on the inhibition of protein synthesis caused in HGMVECs to study if it might be a therapeutical option for patients who are already infected with the toxin.

**Figure 6:**
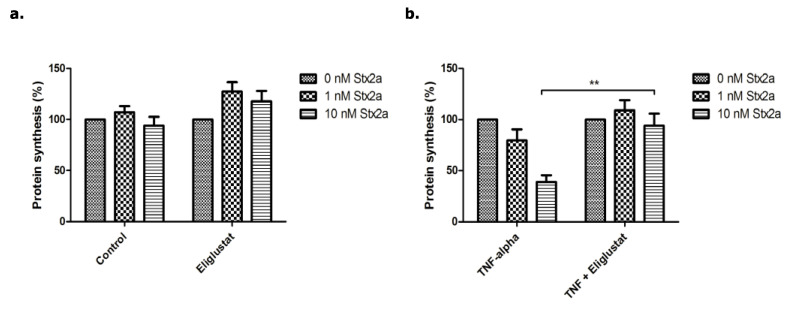
Protein synthesis of primary HGMVECs incubated with Eliglustat and Stx2a at the same time for 24 h. (**a**) Protein synthesis of HGMVECs incubated with 8.0 nM Eliglustat and Stx2a at the same time for 24 h. (**b**) Protein synthesis of HGMVECs pre-incubated with 10 ng/mL of TNFα for 24 h and incubated with Eliglustat together with Stx2a simultaneously for 24 h. Statistical significant differences are indicated with double characters (*p*-value < 0.01).

Primary HGMVECs from three different donors were pre-incubated with or without 10 ng/mL TNFα for 24 h. Next, cells were incubated with 8.0 nM Eliglustat together with 1.0 nM or 10 nM Stx2a for 24 h simultaneously, and protein synthesis was determined (Figure 6a). Pre-incubation of HGMVECs with TNFα and incubation of cells with Eliglustat in combination with 10 nM Stx2a resulted in a clear significant decrease in protein synthesis inhibition (6% protein synthesis versus 61% in TNFα alone) (Figure 6b). In conclusion, Eliglustat also had a clear protective effect against protein synthesis inhibition caused by Stx2a when simultaneously given with the toxin.

### 3.3. The Effect of Eliglustat and Agalsidase Alpha on Cellular Gb3 and Ceramide Levels

As Agalsidase alpha showed only minor effects on Stx-B binding to the endothelial cell surface and protein synthesis inhibition caused by Stx2a, we examined the effect on cellular Gb3 and ceramide levels for both compounds. It might be the case that Agalsidase alpha has no effect on cell surface Gb3 levels, but still influences the amount of Gb3 present in the cell. In addition, lactosylceramide and glucosylceramide were measured.

Primary HGMVECs from two different donors were incubated with or without TNFα, Eliglustat, or Agalsidase alpha for 24 h. Cellular Gb3, lactosylceramide, glucosylceramide, and ceramide levels of the isoforms d18:1/X and d18:2/X were determined with LC-MS. The different glycosphingolipid species were measured after deacylation and did not contain a fatty acid anymore.

Incubation of cells with 10 ng/mL TNFα for 24 h increased the amount of cellular Gb3 when compared to the control group (Table 1). Decreased cellular Gb3 levels were found when HGMVECs were incubated with Eliglustat, Agalsidase alpha, or TNFα + Eliglustat in comparison to the control group (Table 1). Cellular ceramide levels were minimally increased in the TNFα alone and TNFα + Eliglustat cells, while slightly decreased levels were measured when HGMVECs were incubated with Eliglustat or Agalsidase alpha alone and compared to the control cells (Table 1).

Lastly, BOECs from two different donors were incubated with TNFα alone or TNFα in combination with Eliglustat for 24 h. TNFα increased the amount of cellular Gb3 in comparison to the control cells (Table 1), while decreased cellular Gb3 levels were measured when cells were incubated with TNFα + Eliglustat vs. the control cells (Table 1). Minor decreased ceramide levels in BOECs were observed when incubated with TNFα alone or TNFα + Eliglustat and compared to the control cells (Table 1).

## 4. Discussion

In this study, the ability to target the glycosphingolipid pathway as an indirect way to influence Gb3 and the sensitivity of human endothelial cells for Stx2a was investigated. Eliglustat is known as inhibitor of glucosylceramide synthase, which inhibits the formation of lactosylceramide, the precursor of Gb3, while Agalsidase alpha, replacing alpha-galactosidase activity, stimulates the lysosomal breakdown of cellular Gb3 [21,23]. Eliglustat decreased the Stx-B binding to the cell surface of both primary HGMVECs and BOECs pre-stimulated with and without the pro-inflammatory cytokine TNFα. In addition, and more importantly, Eliglustat protected endothelial cells against the inhibition of protein synthesis when used simultaneously with Stx2a. These effects were not seen with Agalsidase alpha. Finally, Eliglustat and Agalsidase alpha both decreased the amount of cellular Gb3 (isoforms d18:1/X and d18:2/X) of primary HGMVECs and BOECs.

The difference between Eliglustat and Agalsidase alpha on Stx-B binding might be explained by the fact that Agalsidase alpha is effective in the lysosome, while in contrast, Eliglustat operates in the Golgi apparatus. Eliglustat showed minor effects on ceramide levels, which can be clarified by the fact that glucosylceramide synthase (inhibited by Eliglustat) and alpha-galactosidase (substituted by Agalsidase alpha) are active after the generation of ceramide.

In the past, Garimano et al. [25] incubated human colonic epithelial cells with Stx in combination with and without Eliglustat [25]. In line with our results, they observed a decreased entry of the toxin and a decreased cytotoxic effect on cells incubated with Eliglustat [25]. Silberstein et al. [35] investigated the effect of C-9 on human proximal tubular epithelial cells after Stx incubation. C-9, known as a potent inhibitor of glucosylceramide synthase, prevented the tubular cells from the cytotoxic effect of Stx by decreasing Gb3 levels [35]. The same group evaluated the effect of C-9 on rats injected with Stx and oral treatment with this compound reduced renal and intestinal injuries with 50% [36]. Recently, Sanchez et al. [26] published the protective effect of Eliglustat against Stx2 in human renal tubular epithelial cells [26]. Pre-treatment with Eliglustat reduced Gb3 expression and prevented the effect of Stx2 on cell viability, proliferation, and apoptosis [26].

Abe et al. [37] tested the inhibitory effect of d-*threo*-phenyl-2-decanooylamino-3-morpholino-1-propanol (PDMP) prototypes on glucosylceramide synthase in Fabry lymphocytes. Depletion of Gb3 with 70 to 80% was measured and binding of Stx-B clearly decreased [37]. In contrast to primary HGMVECs and BOECs, lymphocytes derived from Fabry patients have accumulated Gb3 and, with this in mind, it is hard to compare our results with results from this study. However, Eliglustat is not the only SRT available for patients with lipid storage disorders. Girard et al. [38] showed the protective effect of Miglustat against Stx in human glomerular endothelial cells and proximal tubular epithelial cells. Reduced cell death, intracellular edema, and cell detachment were noticed and a decrease in Gb3 expression was shown. Interestingly, pre-treatment with Miglustat for 48 h caused a significant protection compared to 24 h of treatment [38]. As Miglustat and Eliglustat both inhibit glucosylceramide synthase, it is expected that Miglustat has similar effects on primary HGMVECs and BOECs. Both compounds are FDA approved for the treatment of glycosphingolipid storage disorders and might be good treatment options for STEC-HUS; however, the reported side effects of both compounds makes Eliglustat probably more favorable for clinical use in patients with STEC-HUS [39,40].

In contrast to the studies described with Eliglustat above, the in vitro experiments in our study were carried out with primary human glomerular endothelial cells. As (glomerular) endothelial damage is seen as one of the central hallmarks of the thrombotic microangiopathy seen in STEC-HUS, it is of great value to understand the effects of Eliglustat in combination with Stx2a for the human glomerular endothelium. Primary isolated glomerular endothelial cells were used, which have the advantage of not being genetically altered and are the closest representative for one of the main cell types involved and damaged in HUS patients. Lastly, cells were activated with the cytokine TNFα, a pro-inflammatory cytokine present during disease activity, and the combination of TNFα and Stx2a more closely reflects the actual situation during the acute phase of disease [41].

From a pathophysiological point of view, studying the effect of Eliglustat in the upcoming 3D glomerular on a chip model might give us a better understanding of the cellular interactions under dynamic conditions of the various glomerular cells and bring us closer to the in vivo human situation.

The best prevention for getting STEC-HUS is to avoid intake of STEC-contaminated products, but this is clearly impossible. A low inoculum of STEC can already lead to STEC-infection, STEC-induced bloody diarrhea, and HUS [42,43]. However, it might be possible to inhibit or decrease the effect of Stx on the glomerular cells, tubular cells, and podocytes in the kidney once the toxin is present in the circulation. The observations in this study and recent publications by others [25,26,35,36] strengthen the hypothesis that inhibition of glucosylceramide synthase and thereby blocking the formation of Gb3 may be a therapeutic strategy for future purposes. Eliglustat, given orally, has been approved by the FDA in 2014 and is already on the market for the treatment of patients with Gaucher disease [23,24]. It is metabolized in the liver and not in the kidney. No dosage adjustments have to be made in patients with kidney failure. It has the advantage that IC50 values in humans and its mild side effects like fatigue, headache, and back pain are already known [22]. Glucosylceramide synthase inhibitors such as Eliglustat could be therapeutical for STEC-infected patients with bloody diarrhea, who might develop HUS. In addition, it might be a future therapeutic to prevent renal damage in those who have already developed STEC-HUS.

## Figures and Tables

**Table 1:** Cellular Gb3, lactosylceramide, glucosylceramide, and ceramide levels of primary HGMVECs and BOECs. HGMVECs and BOECs were incubated with or without 10 ng/mL of TNFα, 8.0 nM of Eliglustat, 10 μg/mL of Agalsidase alpha, or TNFα together with Eliglustat for 24 h. Cellular Gb3, lactosylceramide, glucosylceramide, and ceramide levels of the isoforms d18:1/X and d18:2/X were determined with liquid chromatography-mass spectrometry (LC-MS). Levels of the different donors (N = 2 for HGMVECs and N = 2 for BOECs) and two different isoforms d18:1/X and d18:2/X were averaged and standardized to the control group (100%). Gb3 = Globotriaosylceramide; LacCer = Lactosylceramide; GlcCer = Glucosylceramide; Cer = Ceramide; n.a. = Not analyzed.

	**HGMVECs**			
	**Gb3 (%)**	**LacCer (%)**	**GlcCer (%)**	**Cer (%)**
Control	100	100	100	100
TNF-alpha	172	89	128	116
Eliglustat	74	59	49	88
Agalsidase alpha	64	68	75	93
TNF + Eliglustat	61	56	51	110
	**BOECs**			
	**Gb3 (%)**	**LacCer (%)**	**GlcCer (%)**	**Cer (%)**
Control	100	100	100	100
TNF-alpha	177	93	184	80
Eliglustat	n.a	n.a	n.a.	n.a.
Agalsidase alpha	n.a	n.a.	n.a.	n.a.
TNF + Eliglustat	79	65	84	91

## Data Availability

Not applicable.
